# An Internal Model Architecture for Novelty Detection: Implications for Cerebellar and Collicular Roles in Sensory Processing

**DOI:** 10.1371/journal.pone.0044560

**Published:** 2012-09-05

**Authors:** Sean R. Anderson, John Porrill, Martin J. Pearson, Anthony G. Pipe, Tony J. Prescott, Paul Dean

**Affiliations:** 1 Department of Psychology, University of Sheffield, Sheffield, United Kingdom; 2 Bristol Robotics Laboratory, Bristol, United Kingdom; National Microelectronics Center, Spain

## Abstract

The cerebellum is thought to implement internal models for sensory prediction, but details of the underlying circuitry are currently obscure. We therefore investigated a specific example of internal-model based sensory prediction, namely detection of whisker contacts during whisking. Inputs from the vibrissae in rats can be affected by signals generated by whisker movement, a phenomenon also observable in whisking robots. Robot novelty-detection can be improved by adaptive noise-cancellation, in which an adaptive filter learns a forward model of the whisker plant that allows the sensory effects of whisking to be predicted and thus subtracted from the noisy sensory input. However, the forward model only uses information from an efference copy of the whisking commands. Here we show that the addition of sensory information from the whiskers allows the adaptive filter to learn a more complex internal model that performs more robustly than the forward model, particularly when the whisking-induced interference has a periodic structure. We then propose a neural equivalent of the circuitry required for adaptive novelty-detection in the robot, in which the role of the adaptive filter is carried out by the cerebellum, with the comparison of its output (an estimate of the self-induced interference) and the original vibrissal signal occurring in the superior colliculus, a structure noted for its central role in novelty detection. This proposal makes a specific prediction concerning the whisker-related functions of a region in cerebellar cortical zone A_2_ that in rats receives climbing fibre input from the superior colliculus (via the inferior olive). This region has not been observed in non-whisking animals such as cats and primates, and its functional role in vibrissal processing has hitherto remained mysterious. Further investigation of this system may throw light on how cerebellar-based internal models could be used in broader sensory, motor and cognitive contexts.

## Introduction

The idea that internal models are used for sensorimotor processing (e.g. [1]) is of great current interest, particularly in the context of cerebellar function (e.g. [2], [3], [4]). A major theme is the potential role of the cerebellum in predicting future sensory signals, predictions that could be used in a wide variety of sensory, motor and possibly cognitive contexts [2], [5], [6]. But at present there is little detailed information about how workable algorithms could be implemented by known anatomical circuitry to enable the cerebellum to play such a role. To address this issue, we investigated how an internal-model based algorithm could be used to improve detection of novel sensory stimuli, and whether the circuitry required to implement the algorithm has a plausible anatomical counterpart.

Novelty detection is a relatively simple but important example of a generic problem in active sensing. However, the task of novelty detection is hindered when the animal's own movements generate signals in the sensor independently of any changes in the external world. In these circumstances sensory input becomes a mixture of self-produced (‘reafferent’) and externally produced (‘exafferent’) signals (for recent review see [7]). Separating these signals is important for many different purposes (e.g. [8]. For example, when active sensing is used to characterise features of the environment such as surface texture, then characteristics of sensor movement such as its speed must be related to features of the input signal. In other circumstances however reafferent signals can be regarded simply as noise or interference that needs to be removed in order to reveal external events. This is particularly true for what we term here ‘novelty detection’. Unexpected sensory input is of great biological significance, inasmuch as it may be signalling either immediate danger or the presence of prey. It is therefore very important for detection of these signals not to be impaired by interference from the animal's own movements.

The specific example chosen for investigation was the detection of novel whisker contacts during exploratory whisker movements (whisking). Although it is well-known that active whisking has computational advantages for vibrissal processing, it also has a potential disadvantage in producing reafferent whisker signals that could interfere with novelty-detection [9], [10], [11]. Reafferent signals in the vibrissal system have been observed since some of the earliest investigations into active whisking [12] and continue to generate interest [13], [14], [15]. The presence of these reafferent signals immediately raises the notion that an internal model/cerebellar-based novelty detection scheme might be of benefit to vibrissal processing. Imaging and clinical studies indicate that the cerebellum is involved in active tactile sensing [16], [17], [18], [19], [20], and the anatomical circuitry underlying the processing of whisker input in rats includes many cerebellar connections [21], [22]. To date, however, there has been limited progress in understanding these vibrissal sensory-motor loops through the cerebellum, to the extent that even hypotheses of these loop functions are virtually non-existent [22], [23], [24]. In addition, whisking robots have been constructed [9], [10], [25], that enable potential detection-algorithms to be evaluated for practicality, so allowing only workable examples as candidates for subsequent neural investigation. These robots offer an alternative to current models of the vibrissal system, which because of difficulties in mathematically describing the effects of whisker contacts, are not yet suitable for studying the problem investigated here [26], [27].

**Figure 1:**
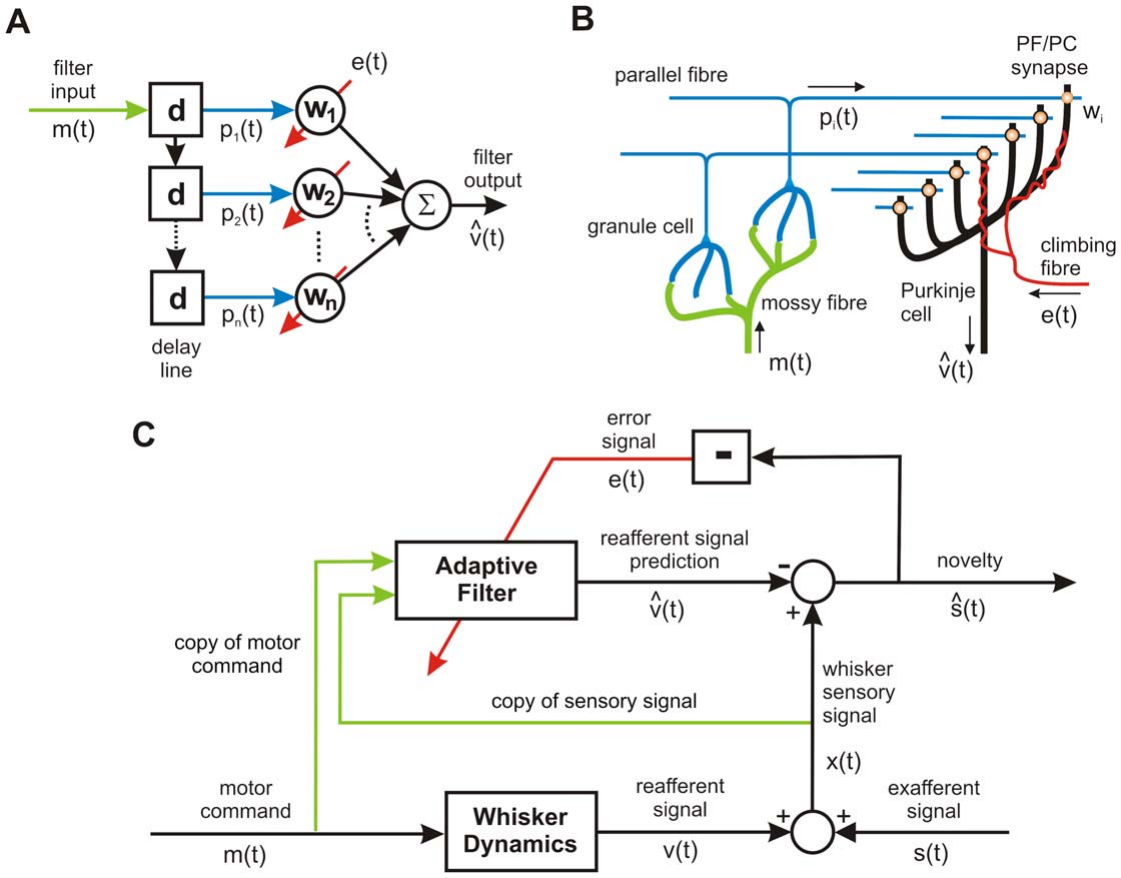
Adaptive-Filter Model of Cerebellar Cortex and Novelty-Detection Architecture. A: The adaptive filter. The input 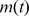 to the analysis-synthesis adaptive filter is passed through a bank of fixed filters (implemented by e.g. tapped delay lines as shown in the diagram) to produce a set of ‘analysed’ signal components 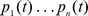 These components are weighted and summed (‘synthesised’) to produce the filter output 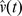. Adaptation of weight 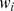 is driven by the correlation between the corresponding component signal 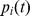 and teaching or error signal 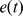. Adapted from Fig 1A of Porrill et al [130]. B: The cerebellar microcircuit. Mossy fibre input 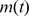
is distributed over many granule cells, whose axons form parallel fibres which synapse on Purkinje cells conveying a set of signals 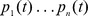. In models of Marr-Albus type the correlated firing of a parallel fibre 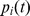 and the single climbing fibre 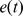 which winds round the Purkinje cell dendritic tree, alters the efficiencies 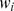 of synapses between parallel fibres and Purkinje cells. Purkinje cell firing 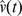 constitutes the output of the microcircuit, and consists primarily of simple spikes assumed to represent the sum of the weighted parallel fibre inputs. Complex-spike firing represents the climbing-fibre input. Adapted from Fig 1A of Porrill et al [130]. C: The novelty detection scheme. The motor command  drives whisker movements, and generates a reafferent signal 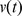 as a consequence. Exafferent signals 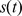 are generated by contact of the whisker with objects. In additive combination, the reafferent and exafferent signals comprise the observed sensory signal 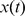. The adaptive filter, which predicts the reafferent signal, is driven by copy of motor commands, the sensory signal, or both. The output of the scheme, the novelty signal 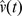, is subtracted from the observed whisker to detect novel events 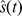 and the negative of this signal drives adaptation of the filter weights via the climbing fibre input as indicated by the box containing the subtraction sign.

The model architecture for improving novelty detection in whisking robots is shown in Fig 1. The cerebellar-based part of the model is the adaptive filter [28], which combines a broad structural resemblance to the cerebellar microcircuit (Fig 1A, B) with proven computational power in signal-processing applications [29]. The adaptive filter is a general-purpose learning device, that can in principle form the adaptive element in the forward, inverse and internal models that have been suggested in the literature (e.g. [30]). Initial application of the adaptive filter to robot whisking [31] used the overall framework of adaptive noise cancellation (e.g. [32], [33]), which deals with the generic problem of removing noise from a signal where some information regarding the noise is available to the system (Fig 1C). In that investigation into robot whisking, it was found that movement of a robot's whiskers generated reafferent signals due to the whisker mechanically affecting the sensors at the base of its own shaft – analogous in principle to reafferent signals observed in rat whisking. The use of the adaptive filter allowed the robot to build an internal model of this process, and so the robot was able to learn to predict the sensory consequences of its own movements and thereby enhance the detection of whisker-object contacts.

In our initial investigation of robot whisking we only used motor-efference copy as input to the adaptive filter for enhancing contact detection. However, the organisation of the rat vibrissal system indicates that the loops through the cerebellum also include whisker sensory signals [22], [23], [24], so these also can be used as inputs to the adaptive filter model. A possible function for these sensory inputs in biological novelty detection is suggested by signal-processing analyses of noise cancellation. A signal correlated with itself over time can be used to predict its own behaviour. This prediction is particularly effective for periodic signals, and can be exploited in the special case of noise cancellation [32]. Since the reafferent signals provided by whisking are likely to contain periodic components (see below) we therefore augmented the adaptive filter with sensory whisking inputs to investigate whether, in principle, such sensory inputs would be of use in an equivalent neural scheme. This would provide a specific computational basis for improving the prediction performance of forward models by the addition of sensory information, a key component for developing a hypothesis of the function of vibrissal sensory-motor loops through the cerebellum.

To complete the investigation we attempted to identify possible neural equivalents to the architecture of Fig 1C, so generating hypotheses for subsequent experimental testing. As noted above, the problem of whisking-induced interference is present in rats: reafferent signals from whisking analogous to those seen in the robot have been observed in the rat trigeminal ganglion [12], [15]. However, how this interference is overcome for the purposes of novelty detection is not well understood. Here we propose that the role of the comparator in the internal-model architecture is carried out by the superior colliculus. This suggestion in turn implies a role for a hitherto functionally mysterious region of the cerebellar cortex, connected to the superior colliculus via the inferior olive. The specification of a particular circuit leads to a number of experimental predictions, and provides a detailed basis for further investigation of how the cerebellum is involved in learning internal models.

## Methods

### Internal models for self-generated noise cancellation and novelty detection

#### Forward model for self-generated noise cancellation

The whisker signals observed by the robot sensors have two components (Fig 1C), those generated by the system's own behaviour (often termed reafferent signals) and those generated by the external world (exafferent signals). In the whisking robot the reafferent signals that are generated by active whisking interfere with the detection of novel events that are transmitted by exafferent signals (contacts). Novelty detection can be improved by the use of a noise cancellation algorithm (see figure 1C), where (i) a filter driven by a copy of motor commands predicts the reafferent component of a sensory signal, and (ii) the prediction is subtracted from the observed sensory signal to highlight novel events (we label a filter driven by copy of motor commands as a forward model).

Formally, assume that a sensory signal 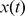, generated by a sensory system, is composed of two additive signals, exafferent 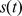 and reafferent 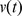,



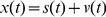
(1)It is important to note that only the sensory signal 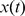 is observed, which is a combination of 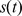 and 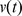. A filter driven by a copy of motor commands can be used to predict the reafferent component. Subtracting the prediction of 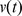
from the observed signal recovers an estimate of the exafferent signal 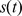, that is,
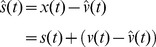
(2)where 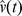 is the filter prediction of the reafferent signal and 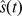
is the output of the novelty detection scheme. Note that when the filter accurately predicts the reafferent signal (i.e., 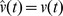) the exafferent signal of interest is completely recovered, 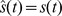.

The key part of the novelty detection scheme is the filter prediction of the reafferent signal. We describe the filter as 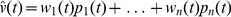
(3)where 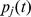 is some transformation of the filter input signal (for instance a delay caused by a linear filter), and 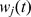
is a filter weight that varies over time. If we assume that the cerebellum implements a computational analogue of this filter (see Introduction), then 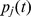
represents parallel fibre signals and 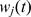
represents parallel fibre-Purkinje cell synaptic weights (see figures 1A and 1B).

The basic requirement of the adaptive filter is that the basis of filters used is able to represent the forward model to sufficient accuracy. In signal-processing a bank of tap-delay line filters is often used to provide the required time-resolution and delay. This representation is not biologically plausible, but computationally equivalent and more biologically plausible linear filters have been proposed [29]. How this re-coding is computed from mossy fibre inputs in the granule cell layer is matter for current debate, but the large scale model of Medina et al. [34] shows that this is possible in principle, and a more recent detailed model of Honda et al. [35] has discussed relevant cellular mechanisms.

Adaptation of filter weights is required in order to learn the dynamic processes that generate the reafferent signals. For the case where filter input is a copy of motor command our previous work has shown that a correlational learning rule can be used to drive adaptation of the filter weights, where the weight update rule is
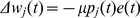
(4)


Here weight update is driven by the correlation between the transformed filter input 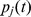 and the error signal 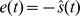 which, in the cerebellar context, correspond to parallel fibre and climbing fibre signals respectively; 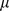 is a scaling parameter that effects the rate of learning. The weight update rule is equivalent to the least-mean-squares (LMS) rule for which convergence properties are well-known [33]. The negative sign in (4) has been chosen to agree with the biological learning rule in which positive correlation between these two signals at the synapses between parallel fibres and Purkinje cells produces depression of the synaptic weight. Hence this scheme predicts that in biological systems the climbing fibres have to transmit a copy of the negative novelty detection scheme output. The mean firing rate of the climbing fibre is typically around 1 Hz. In the adaptive filter model we do not constrain the firing of the climbing fibre to this rate because computationally this would only cause a reduction in learning rate and would not affect eventual convergence in the long-term (cf. [36]).

#### Direct use of sensory signals in novelty detection

The forward model novelty detection scheme discussed above predicts the reafferent signal using a copy of motor command as input. From the noise cancellation literature we know that alternative filter inputs can be used in certain scenarios: for instance in the case of periodic reafferent signals we can drive the filter with a copy of the observed sensory signal 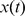 [32]. (A noise cancellation scheme driven by a sensory signal with a periodic reafferent component also requires a delay in the filter that is sufficient to remove predictable components of the exafferent signal in order to obtain unbiased filter weight estimates). The filter then learns to represent the characteristics of the reafferent signal itself, rather than the dynamic process that generates reafference (as in the case of the forward model). Hence we label the novelty detection scheme driven by observed sensory signals as a ‘signal’ model.

For non-periodic reafferent signals this approach would not lead to an optimal solution. However, if we assume that external events are infrequent relative to movements that cause reafferent signals and are also different in terms of signal statistics, then it is likely that use of the sensory signal as input to the filter would improve novelty detection.

#### Sensorimotor integration for enhanced novelty detection

We will show that the predictions of either the forward model or the signal model lead to more effective novelty detection depending on the circumstance: for instance, if the sensory signals are stochastic we would expect to see the forward model outperform the signal model because use of the motor signals facilitate accurate prediction of the whisker motion regardless of the nature of that motion (stochastic or predictable), whereas the signal model relies on the predictable nature of the signal. In an alternative case, if the process generating the sensory signal is of a nonlinear nature, then the (linear) signal model should outperform the (linear) forward model because the signal model is not required to describe the dynamics of the generating process but rather just the structure in the reafferent component of the sensory signal itself (which could be as simple as a sine wave even if the dynamical process generating the signal is highly complicated and nonlinear).

Our hypothesis is that use of both copy of motor commands and sensory signals as input to the predictive filter would improve novelty detection across a variety of movement scenarios and hence ensure robustness. Therefore, in this investigation we use both motor commands and sensory signals as input to the filter in order to form a combined sensorimotor prediction of the reafferent signal (see figure 1C).

#### SCRATCHbot – a physical model of the rat

**Figure 2:**
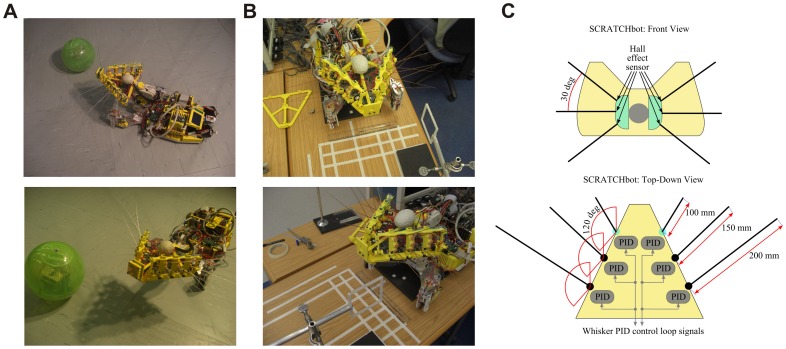
The whisking robot SCRATCHbot. A. Photographs of the mobile whiskered robot SCRATCHbot exploring the environment using its bilateral array of active artificial whiskers. It has been used to test hypothetical models of whisker array based object detection (in this case a sphere) and action selection mechanisms. B. SCRATCHbot in a ‘head-fixed’ preparation on a workbench. Band-pass filtered white noise and periodic whisking patterns were used to drive one of the whisker column actuators that generated the whisker response data sets described here. Upper panel from Fig 3 of ref [31]. C. Top: Front view of robot head, showing two columns of whiskers. There are three rows of whiskers on each side of the head, separated in the frontal plane by 30 deg. Deflections of the whisker shafts caused by reafferent (self-motion) and exafferent (contact) stimuli are measured at the base using IC tri-axis Hall effect sensors. Bottom: Top-down view of robot head. Six columns of whiskers are actuated independently by DC motors under Proportional, Integral, Derivative (PID) control through a maximum 120 deg. of rotation. The lengths of the whiskers decrease from front to back of the head (100–200 mm). From Fig 4 of ref [31].

Experimental whisking data was generated using a biomimetic whiskered robot, which functioned as a physical model of the rat whisker system. The whisking robot, called Spatial Cognition and Representation Through Active TouCH robot or SCRATCHbot for short (Fig 2), represents the second generation of a series of whisking robots developed by the Bristol Robotics and Sheffield Biotact Laboratories [11], [27]. SCRATCHbot has 18 whiskers (made from plastic), arranged on each side of the head in 3 by 3 arrays (9 on each side). Similarly to the rat, whisker thicknesses and lengths vary across the vibrissal array, with smaller, thinner whiskers located rostrally and longer, thicker whiskers located caudally (further details in [31]). A magnet is bonded to the base of each whisker, in a biomimetic follicle. A Hall effect sensor is used to measure the movements of the magnet in 2-dimensions, from which whisker angle is obtained that was used as sensory input to the adaptive filter. Whiskers in a column are mounted into the same carrier,each of which is independently actuated around the vertical axis of the column by a DC motor. Therefore, all whiskers in a column are actively whisked back and forth in synchrony with each column afforded120 degrees of rotation controlled by a proportional, integral and derivative (PID) controller. Each columnar PID controller tracks a desired reference trajectory (angular position of the whisker carrier) specified by the operator.

**Figure 3:**
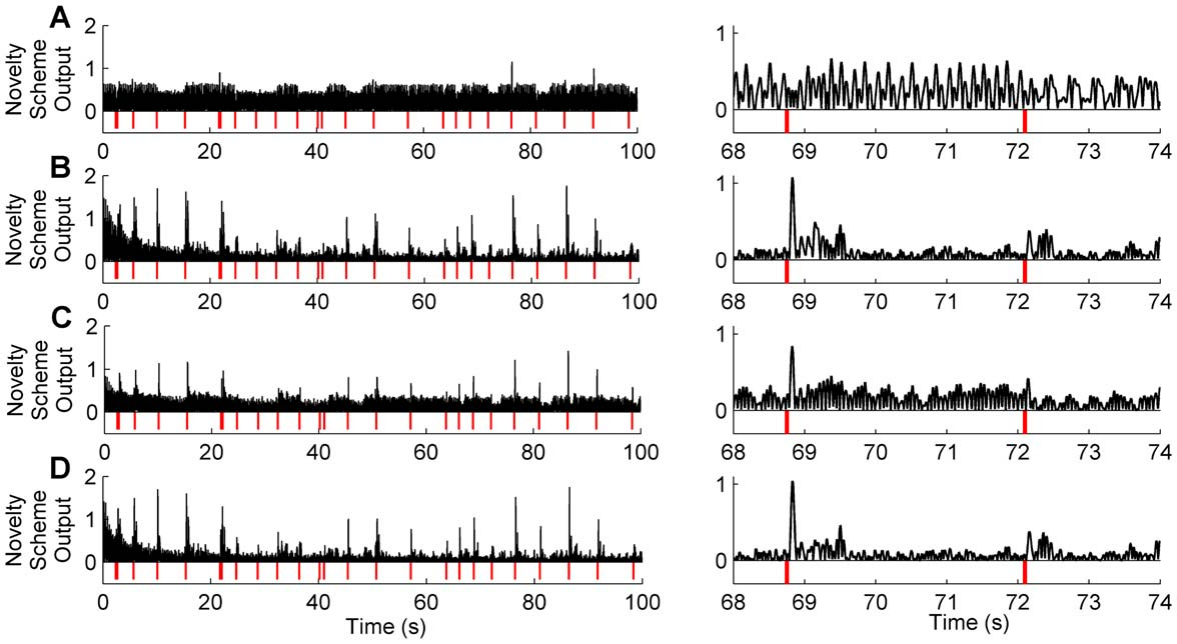
Robot contact detection. Novelty detection algorithms applied to periodic signals recorded from the whisking robot for a 100 s data record (left) and an illustrative section zoomed on the time axis (right). For an effective visual comparison the novelty signals are normalised by the peak value in the final 10 s, and the absolute value is displayed (in black) so that contacts (in red) are clearly marked. A: Raw sensory signal. B: Sensory input only. The initial period of ∼20 s during which the model is learned can be seen clearly as a decrease in background noise. C: Motor input only. D: Sensorimotor input.

#### Experimental data generation

Sensory signals were recorded from the whisking robot for offline analysis. One whisker on the robot was actively moved under head fixed conditions for 100 s, with contacts randomly delivered to the whisker by manually tapping it with a plastic rod. Whisker movement was driven by a periodic wave: a square wave of frequency 3 Hz with a duty cycle of 40%, low-passed filtered with a first order filter (time constant of 60 ms), to produce a sawtooth pattern similar to fast/slow phases of protraction/retraction in rat-whisking. Whisker sensory signals were recorded at a sample rate of 2 kHz and were down-sampled for the offline analysis to 200 Hz (and low-passed filtered at 10 Hz). In addition to contact data, free-whisking (i.e. contact-free) signals were recorded to facilitate the identification of a robot whisking model (discussed below); in this case, whisking input was a ‘rat-like’ signal – a looped version of observed rat-whisking recorded by Towal and Hartman [37] and used in our previous study [31].

### Synthetic Data Model

#### Robot whisking model

In order to study the effects of different whisking types (stochastic and periodic) and process nonlinearities in a more repeatable and controlled way than was possible on the robot platform, we developed a dynamic model of robot whisking: a reafferent model 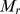
driven by motor commands and an exafferent model 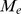 driven by simulated contacts. The reafferent model was obtained using standard system identification techniques (minimisation of model prediction error using least-squares) applied to the free-whisking signals recorded from the robot (low-pass filtered at 5 Hz in order to focus on the low frequency linear input-output dynamics of whisking) in response to motor commands derived from whisking patterns observed in the rat [38]A second order discrete-time linear transfer function was used to model the input-output dynamics of robot whisking (motor commands as input, reafferent sensory signals as output),
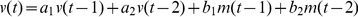
(5)where 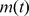 was the motor command input.

An impulse response filter was used to represent the robot contacts (a model of exafferent responses) where the contact input was defined as an impulse 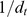, where 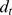 was the sample time, and the filter output was the exafferent signal 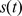. A pair of typical whisking contacts was selected from recorded signals and the contact responses, which were not directly measured, were defined as the difference between the prediction of the reafferent model defined in eqn (5) and the observed sensory signal. The impulse response model was fitted to these inferred contact signals.

The two robot model components were used to generate simulations of reafferent and exafferent signals, which were combined additively as described in eqn (1) to produce the combined sensory signal. The robot whisking model was used to generate simulated experimental data consisting of multiple trials of long time duration, with precise and repeatable control of whisker contacts – a feature not readily possible to achieve in the laboratory on the actual robot.

The robot whisking model was used to study two features: (i) whisking modes (stochastic versus periodic) and (ii) system nonlinearities. The stochastic whisking input was defined as band-pass filtered Gaussian distributed white noise (with pass-band between 2 and 4 Hz) and the periodic signal was defined as a sine wave of frequency 3 Hz. The process nonlinearity was introduced to the input-output model in eqn (5) as an additive bilinear cross-product term between motor input and reafferent signal output 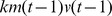. The coefficient 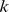 was varied to produce weak-to-strong nonlinear effects. This is a generic non-linearity representing a form of state-dependent input gain (e.g. [39], [40]). Its simplicity makes it a good candidate for testing the hypothesis that novelty detection can be improved by using a signal model when nonlinearities are present. The nonlinearity also produces harmonics in the output which would be typical of a vibrating structure such as a cantilever beam driven by a forcing input, analogous in certain respects to a whisker [41].

Each simulated experiment was run for a duration of 10^3^ seconds. Contacts were stochastically generated in that time window by drawing a sample from a uniform random distribution (between zero and one) at each sample time and specifying occurrence of a contact if the sample value exceeded a threshold (set to 0.999 so that contacts were relatively infrequent). Each simulation was repeated 20 times, in order to demonstrate the consistency of novelty detection results.

#### Novelty detection

We implemented the novelty detection schemes in Matlab. Filter predictions of the sensory consequences of movement were obtained by implementing eqn (3), with weight adaptation at each sample time obtained by eqn (4), and the filter was driven with three separate input types: (i) copy of motor commands, (ii) observed sensory signal and (iii) both copy of motor commands and observed sensory signals. The output of the novelty detection scheme was obtained using eqn (2). Adaptive filter weights were initialised to zero. Filter input signals were transformed by a tap-delay line, adjusted to a length of 100 taps for motor-only and sensory-only inputs, and 200 taps for sensorimotor inputs (100 taps for each input type: 5 ms between taps). The learning rate parameter 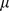 was set to 0.01 (i.e. relatively fast adaptation) when processing the short time duration physical experimental data and 5×10^−4^ (relatively slow adaptation) when processing the much longer duration simulated experiments. In order to more closely mimic the biological limitations n of the novelty detection schemes, we introduced a small delay into the filter processing of 10 ms.

We use a basic novelty detection scheme in which a contact is signalled when the relevant signal crosses some detection threshold. The metric used for analysing performance of the novelty detection schemes was improvement in signal-to-noise ratio (SNR) over the baseline raw signal, where SNR in decibels is
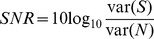
(6)


This quantity is directly related to the ease with which a threshold can be set to reliably distinguish true contacts from threshold crossings due to self-motion. We defined the ‘signal’ in the SNR quantity as the variance of the novelty detection scheme output 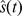
for a 200 ms window post-contact (because it is this exafferent ‘contact’ signal we were seeking to enhance). The ‘noise’ in the SNR quantity was defined from the variance of the remaining portion of the novelty detection scheme output (the reafferent signal). The SNR was only obtained from the final 200 s of the simulation, so that the results were not distorted by the significant weight adaptation taking place in the initial phase of the experimental trial.

## Results

### Overview

In previous work we have investigated how far cancellation of reafferent whisker signals can be achieved by driving the adaptive filter model of cerebellum with copies of motor commands. Here we extend that investigation by making use of the fact that vibrissal loops through the cerebellum also include whisker sensory signals themselves, so providing a second type of input to the adaptive filter model. Therefore, in order to provide computational evidence for why such sensory inputs would be of use in novelty detection, we focused on comparing the performance of novelty detection schemes with sensory, motor and sensorimotor inputs to the adaptive filter. In order to explore and compare the effectiveness of different novelty detection schemes we used experimental data from a robot rat and also a simulation model based on the robot. The advantage of this combined approach was that we were able to demonstrate the principle of novelty detection on actual robot data as a physical model of the vibrissal system, and then provide a deeper exploration of different whisking scenarios in a more controlled *in silico* environment.

### Novelty Detection in Robot Rat

#### Experimental Data

During normal operation of the whisking robot, we observed reafferent components in the whisker sensory signal. This reafferent signal interfered with the detection of contacts, impairing the operation of the robot: contacts were either missed if the contact detection threshold was raised too high, or false contacts were generated if the threshold was set too low. Therefore, in order to demonstrate the benefits of the three novelty detection algorithms investigated here (using sensory-only, motor-only or sensorimotor input to the adaptive filter) we first present a comparison of the algorithms applied to the periodic robotic whisking signal, where it is strikingly apparent from visual inspection that the output from any of the novelty-detection algorithms is much more useful than the raw sensory signal for the purpose of contact detection (see figure 3).

#### Simulated Data

**Figure 4:**
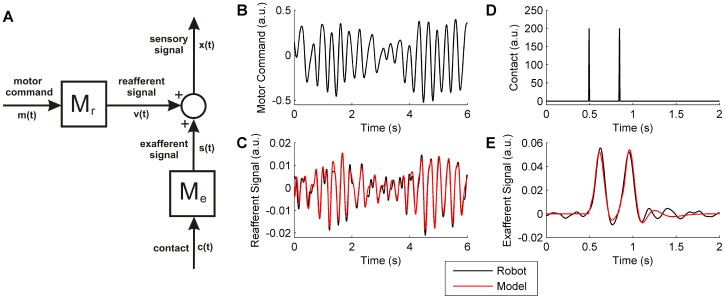
Robot simulation. Description of the robot model used in simulation studies and results of modelling. A: Model description: the simulated sensory signal 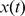 is obtained from the sum of two models, a reafferent model 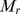, driven by motor commands and an exafferent model 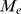, driven by contacts. The dynamics models 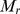 and 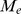 were identified as linear systems from the pre-processed experimental robot data. In order to investigate additional dynamic nonlinearities in the motor-reafferent pathway, the model 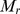 was modified to include a bilinear term, which gave rise to harmonics in the reafferent signal. B: Reafferent model input signal (motor command), based on data from rat whisking [38] Analysis of this signal indicates it to be approximately periodic with a narrow-band Fourier spectrum. [31] C: Comparison of the robot free-whisking signal with the reafferent model prediction shows excellent agreement. D: Exafferent model input signal (impulses). E: Comparison of the robot contact signal with the exafferent model prediction shows excellent agreement.

In order to investigate the novelty detection schemes in a systematic way not possible in the laboratory we developed a model of robot whisking. The robot whisker dynamics were modelled using system identification methods (described in METHODS). We first verified that the identified model accurately represented the dynamic behaviour of the robot (comparison of model predictions to robot data are given in figure 4) in response to motor commands based on data from rat whisking [31], [38]. The model was then used to generate multiple trials of robot whisking, for both stochastic and periodic whisker movements, and also simulations with additional nonlinear dynamics.

**Figure 5:**
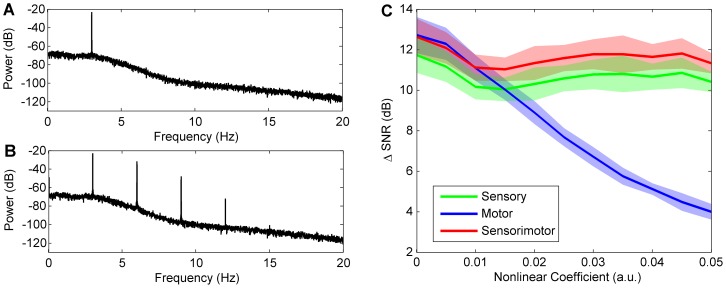
Plant nonlinearity. Effects of plant nonlinearity on novelty detection for periodic whisking (3 Hz), using a model of the robot plant. A: Power spectrum of the sensory signal for the linear case, where the bilinear term coefficient *k* (a measure of plant nonlinearity described in Methods), was set to 0. The model was excited with an input sine wave of frequency 3 Hz, which produced the clear peak at 3 Hz in the power spectrum of the reafferent output signal. Due to the linear dynamics, the system only responded at this frequency. B: Power spectrum of the sensory signal for the strongest nonlinear case tested here, where the bilinear term coefficient 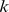 was set to 0.05. Note the appearance of harmonics (at 6, 9, 12, 15 Hz) that are absent from the linear simulation signal shown in A, which is an effect of including the nonlinear dynamics. C: Performance of each novelty detection scheme in terms of improvement in signal-to-noise ratio (SNR) over the baseline raw signal, when varying the nonlinear coefficient 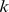 of the bilinear term from 0 to 0.05. Mean performance of 20 trials is shown as the solid line and standard deviation is shown by the shaded region.

We have demonstrated previously the effectiveness of using a forward model (i.e. motor-only input) for novelty detection in a linear dynamical context, with non-periodic whisking [31]. Here we show the degradation in performance of the forward model novelty detector as the dynamics of the reafferent generating process are made increasingly nonlinear (linear and nonlinear signals are contrasted in figures 5A and 5B). The poor performance of the forward model scheme contrasts to the novelty detector driven by sensory input for the case of periodic signals, where the use of the sensory signal is particularly effective even in the case of nonlinear dynamics (see figure 5C). The improvement in signal-to-noise ratio (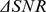: see Methods) resulting from the use of the novelty detection algorithms is apparent for all methods and a high level of improvement is maintained for varying strengths of nonlinearity in both the sensory-only and sensorimotor cases (see figure 5C). However, the novelty detector that uses motor-input only (i.e. the forward model) falls off in performance as the strength of nonlinearity is increased, towards a level that is only just above the raw signal itself (see figure 5C).

**Figure 6:**
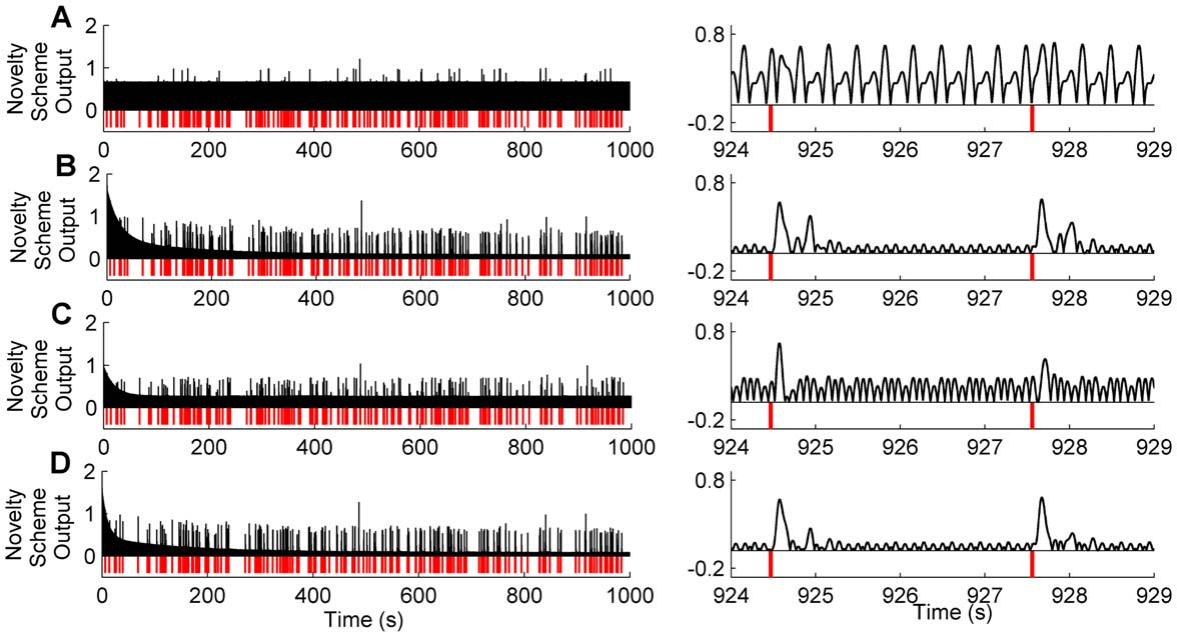
Periodic Signals. Novelty detection algorithms applied to periodic signals generated by simulation of the robot model, for a 1000 s data record (left) and an illustrative section zoomed on the time axis (right). For an effective visual comparison the novelty signals are normalised by the peak value in the final 200 s, and the absolute value is displayed (in black) so that contacts (in red) can be clearly marked below the output. A: Raw signal. B: Sensory input only. C: Motor input only. D: Sensorimotor input.

An example of one experimental trial corresponding to the results in figure 5C is shown in figure 6, for the case where the nonlinearity is strongest (the nonlinear term coefficient 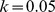). Inspection of figure 6 demonstrates the clear improvement in the ability to distinguish novel events from the novelty detection scheme outputs compared to the raw signal, especially in the schemes that use the sensory signal in the filter input.

**Figure 7:**
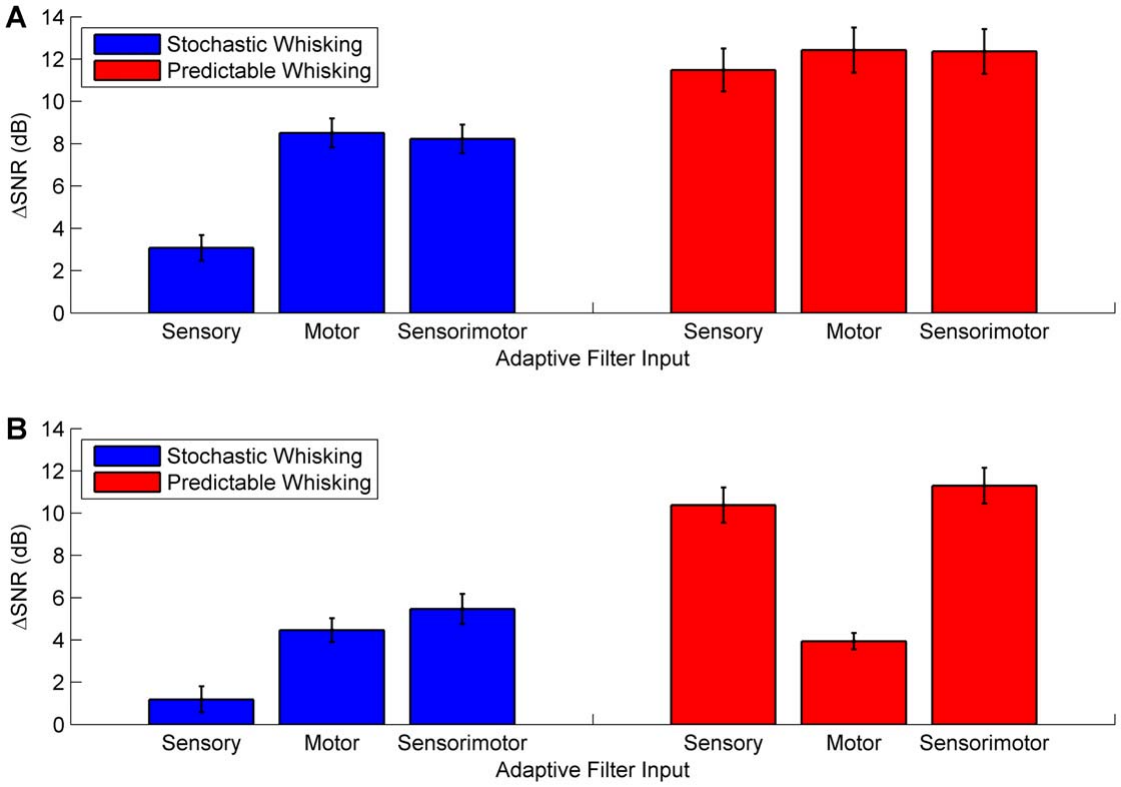
Summary of algorithm performance. Comparison of novelty detection schemes when (i) either whisking stochastically or periodically and (ii) when the robot model of whisker movement is linear or nonlinear. **A**: Linear model of robot whisking. The bilinear term coefficient, 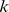 (described in Methods) was set to 0. **B**: Nonlinear model of robot whisking. The bilinear term coefficient, 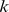 was set to 0.05. Mean level of improvement in signal-to-noise ratio (SNR) over baseline raw signal is shown by the bar plot and standard deviation by the error bars (obtained from 20 trials in each case).

The results from simulation studies of novelty detection for nonlinear, periodic whisking described above have revealed a context in which the forward model is less effective than predictive filters driven with sensory signals. In order to more fully assess performance in various conditions we ran simulations where (i) the whisking was either stochastic or periodic, and (ii) the whisking dynamics were either linear or nonlinear. For these four scenarios we found the following (see figure 7):

1. Stochastic whisking and linear dynamics: The sensory-only novelty detection scheme improved SNR over the raw signal. Motor-only and sensorimotor performed significantly better than sensory-only and similarly to each other.
2. Predictable whisking and linear dynamics: There was an overall improvement in SNR compared to stochastic whisking. The novelty detection schemes gave more similar performance with a marked relative improvement in the sensory-only scheme.
3. Stochastic whisking and nonlinear dynamics: There was an overall drop in performance compared to the equivalent linear case. Sensory-only performance was worst of the three schemes, and sensorimotor performed better than motor-only by a small margin.
4. Predictable whisking and nonlinear dynamics: Unlike the preceding cases, there was a large relative drop in performance of the motor-only scheme. Sensory-only and sensorimotor performed similarly to each other and much better than in the stochastic/nonlinear case.

In summary, these results indicate that the sensorimotor novelty detection scheme performed consistently well in all tested scenarios, in contrast to the sensory- and motor-only schemes. The reason for the improved performance when using sensory inputs to the adaptive filter, in the case of periodic whisking, is that the adaptive filter only has to learn the signal structure, not the dynamics of the generating process (i.e. the whisker plant). This results in particular improvements in SNR for sensory inputs, in comparison to motor inputs, when the dynamics of the plant are nonlinear because the linear filter cannot fully describe the nonlinear motor-to-sensory transformations. It is plausible that the cerebellum may be able to learn such nonlinear dynamic models, which would improve the performance when using motor inputs, although such an expansion of the model is beyond the scope of this investigation.

### Biological Circuitry

**Figure 8:**
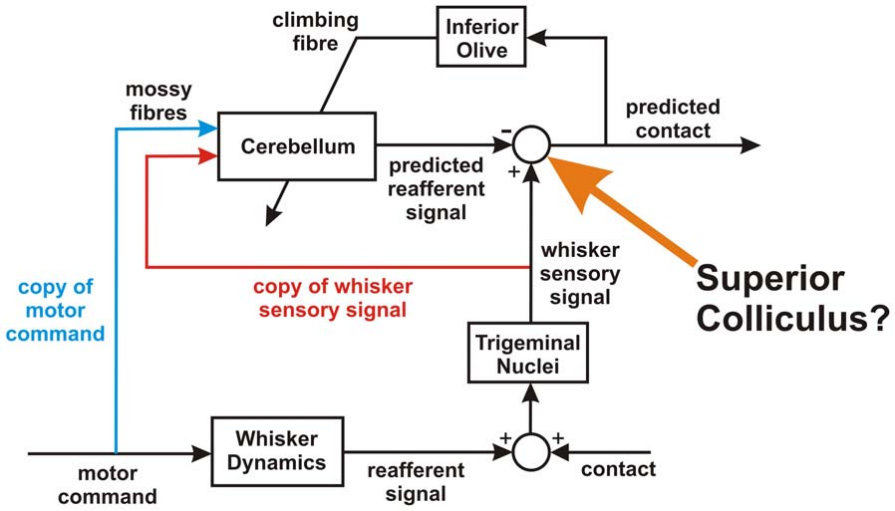
Possible neural equivalent. Possible neural substrate for noise cancellation in the whisking animal. The cerebellum is assumed to correspond to the adaptive filter, and it is proposed that the superior colliculus corresponds to the comparator. This proposal has functional implications for the projections between cerebellum and superior colliculus, and for whisker-related inputs to both structures.

The effectiveness of the signal-processing architecture shown in Fig 1C for improving novelty detection in a whisking robot raises the question of whether it is implemented neurally (Fig 8). If so, an extensive literature (references in [29]) suggests that the cerebellum would be a natural candidate for the adaptive filter (references in Introduction). The implication of Fig 8 is that there should be an area of the cerebellum with the following connections. (i) Two types of mossy fibre input, one carrying sensory information from the whiskers, and the other efference-copy information from whisking commands. Both types of information are needed, because the combination of the two leads to more robust improvements in novelty detection over a range of conditions than either input alone. (ii) Purkinje cell outputs (via the deep cerebellar nuclei) to the comparator. (iii) Whisker-related climbing fibre input (via the inferior olive from the comparator. A central issue then concerns the identity of the neural structure that compares filter output and actual sensory input.

Recent single-unit and imaging data suggest that barrel cortex has a weak response to whisker contact while whisking. Crochet and Petersen [42] made whole-cell membrane potential recordings from barrel cortex neurons in awake mice during whisker-related behaviour. The large depolarizing sensory responses produced by brief passive whisker stimuli were markedly reduced when the animal was whisking. Similar results have been obtained with voltage-sensitive dye imaging of barrel cortex [43], and it has been suggested that the response to unanticipated passive stimuli during whisking is weak because the stimuli "may be confused with self-motion" ([44], p.526).

In contrast, another major recipient of vibrissal information in rodents, the superior colliculus (e.g. [45], [46]) appears to be directly involved in detecting novel whisker contacts.

Initial work on the rodent superior colliculus (reviewed in [47], [48], [49]) indicated that its removal produced a striking visual neglect. This neglect was first interpreted in the context of ‘two visual systems’ [50], later refined [47], [48], [49] to argue that the retinotectal projection in rodents emphasised information about localised transient stimuli particularly in the periphery of the visual field. If the superior colliculus were removed unexpected peripheral stimuli were simply ignored, no matter how interesting, edible or dangerous [51].

However, despite the focus of this work visual processing, evidence was available to indicate that collicular removal produced a vibrissal neglect. Orienting to experimenter-produced vibrissal stimulation is severely impaired by collicular lesions [52], [53], [54], as is orienting to novel environmental features encountered by the vibrissa during free movement in an open field [54], [55]. In addition, direct or indirect activation of the superior colliculus can result in enhanced orienting and biting to vibrissal stimulation [56], [57], and in what appears to be a type of ‘ghost’ orienting in the form of persistent circling and gnawing [58]. This is consistent with more general evidence that the rodent superior colliculus is specialised for mediating a wide variety of responses to unexpected stimuli [51].

Taken together, these pieces of evidence point strongly to the superior colliculus as the central structure for the detection of novel stimuli during whisking. The critical question, therefore, is the extent to which the circuitry of Fig 1C corresponds to specific connections between superior colliculus and cerebellum in the rat. A plausible correspondence in this regard would provide the foundation for a novel hypothesis concerning collicular-cerebellar interactions for detection of novel whisker contacts.

#### Superior Colliculus Projection to Cerebellar Cortex via Inferior Olive

In the internal-model circuit (Fig 1C) the comparator sends a teaching signal back to the adaptive filter. In the adaptive-filter model of the cerebellum (Fig 1A, B), the teaching signal is supplied by the climbing fibre input to Purkinje cells in cerebellar cortex, which originates exclusively from the inferior olive. In the neural equivalent of Fig 1C the rat superior colliculus is therefore shown as projecting to the inferior olive. Abundant anatomical and electrophysiological evidence indicates not only that this projection exists [59], [60], [61], [62], [63], but that it is more extensive than in cats and primates.

**Figure 9:**
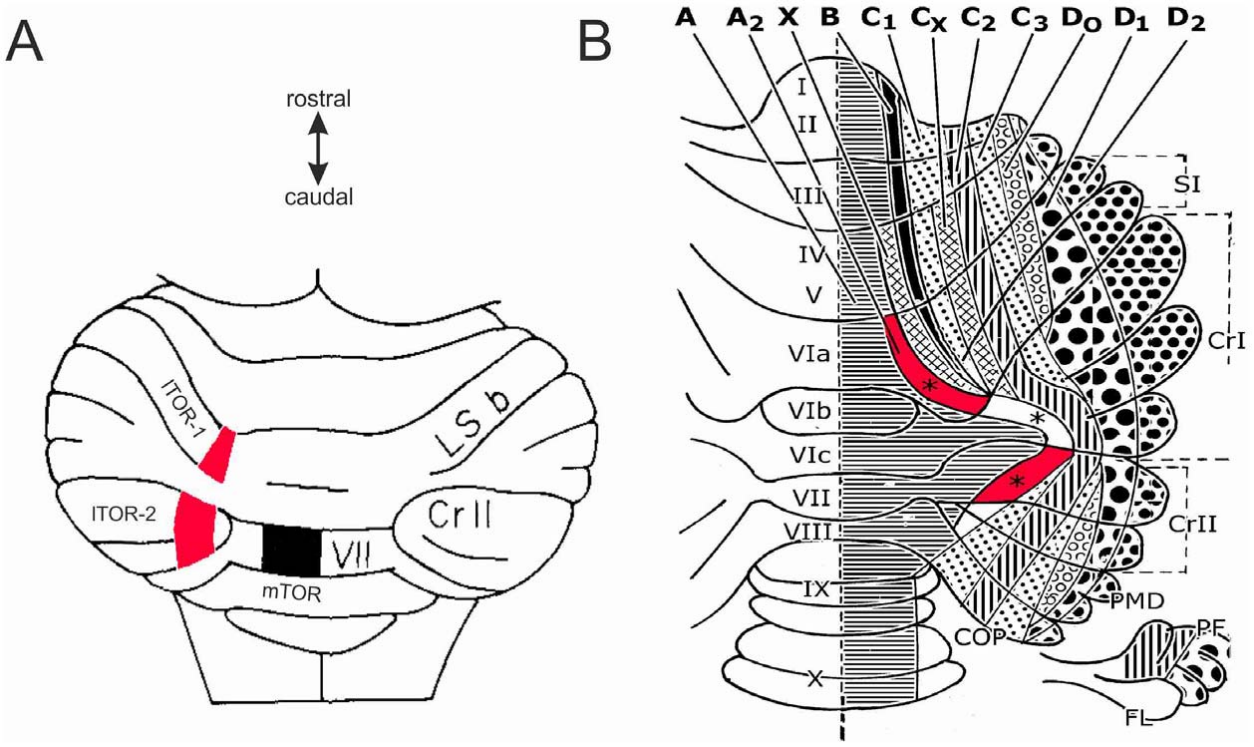
Tecto-olivo-recipient (TOR) areas in rat. A Diagram adapted from Fig 2 of Akaike [100], showing a dorsal view of posterior cerebellum in rat. The medial black area (labelled mTOR) in the vermis of lobule VII corresponds to (part of) the medial tecto-olivary recipient area [64]. The two lateral red areas (labelled lTOR-1and lTOR-2) are both part of the lateral tecto-olivary recipient area, the former in lobulus simplex b (LS-b, part of lobule VI), the latter in crus II (part lobule VII). A third lateral tectorecipient area in the paramedian lobule (also part of lobule VII, immediately caudal to lTOR-2) is referred to but not described in detail by Akaike [63]. B Diagram adapted from Fig 1B of Voogd and Ruigrok [73], in turn adapted from Fig 9 of Buisseret-Delmas and Angaut [67], showing a flattened representation of cerebellar cortex in the rat marked with the locations of parasagittal zones A to D_2_. The lateral TOR areas shown in panel A lie in zone A_2_ [64], and the red patches indicate our estimate of their location. The third lateral TOR area in the paramedian lobule referred to by Akaike would correspond to the caudalmost part of zone A_2_, adjacent to lTOR-2. Subsequent work has shown that this part of A_2_ receives short-latency climbing-fibre and mossy-fibre input from the contralateral face, and that the climbing-fibre input arises from a region of the caudal medial accessory olive [68] apparently similar to that described as receiving projections from the superior colliculus [63].

Thus, the tecto-recipient region of the inferior olive in rats has been found to project to separate areas of cerebellar cortex (Fig 9A), which have been termed the medial and lateral tecto-olivo recipient (TOR) areas by Voogd and Barmack [64]. Cerebellar cortex is organised into parasagittal strips termed zones, each receiving input from olivary cells with similar properties (for references see [65]). The zones are labelled from A (most medial) to D (most lateral) (Fig 9B), and it appears that the medial TOR area is located in zone A1 (lobule VII and possibly part of lobule VI) whereas the lateral TOR area is in zone A2 [66], [67], [68], [69], [70], [71]. The medial TOR area appears similar in location to the oculomotor vermis of cats and primates. In contrast, the lateral TOR areas in zone A2 (the paravermal part of lobules VI and VII) do not have an equivalent in cat or primate, and the olivary cells that project to them are distinct from those that project to the mTOR area [62], [63], [72], [73]. The laterally projecting olivary cells appear to convey vibrissal information, relayed at least in part from the superior colliculus [74], [75].The precise nature of the information is unknown, but recordings from neurons throughout the inferior olive in awake cats have suggested that many "function as somatic event detectors responding particularly reliably to unexpected stimuli" ([76], p.40). This suggestion is consistent in general terms with the role in novelty detection for the lateral tecto-olivo-cerebellar projection.

#### Hypothesis

The success of the signal-processing architecture for model-based novelty-detection in robots suggest it may have a neural counterpart. Given that the superior colliculus is a plausible candidate for the comparator, the issue becomes how far collicular connections with the cerebellum meet the architecture's requirements. Although colliculo-cerebellar loops are known to be important in general terms for vibrissal processing, their specific functions are not known (e.g. [21], [22]). Here we propose that (at least part of) the tecto-recipient zone A2 in rodent cerebellar cortex is involved in detecting novel whisker contacts during whisking. Further details of how far this zone's connections fit with the novelty-detection circuitry are considered in the Discussion.

## Dicussion

### Novelty Detection in Robots

#### Whisking Robot

The computational analysis conducted here has demonstrated that under certain circumstances the additional use of sensory information can improve novelty detection beyond using motor efference copy alone. The benefits of using sensory information were particularly seen where the movements were predictable and the reafferent noise generating process was nonlinear. The use of sensory information was not so beneficial when the whisking was stochastic, hence unpredictable, and the whisking dynamics were linear, although it should be noted that there was always an improvement in SNR over the raw signal regardless of particular novelty scheme configuration (sensory-only, motor-only or sensorimotor).

The sensorimotor novelty detection scheme performed consistently well in all tested scenarios, in contrast to the sensory- and motor-only schemes. The sensorimotor scheme was able to exploit the input signals most useful for the context. For instance, in the stochastic/linear case the sensorimotor scheme made use of the motor information and in the predictable/nonlinear case it made use of the sensory information.

The utilization of different input signals in the novelty detection scheme was automatic, naturally driven by the correlations that existed between filter inputs and error signal, so no prior knowledge of the context was required. Hence, these results suggest that the use of sensorimotor inputs leads to performance that is both effective and robust to changing scenarios – a highly desirable feature of both autonomous robots and biological systems.

#### Comparison with Previous Work on Novelty Detection and Robotics

Typical engineering approaches to novelty detection include statistical methods where a model is constructed from multiple examples of known data in the form of a density function [77]. The model is then used by obtaining the probability that test points originate from the density function, and applying a threshold to determine if they are novel. In particular, techniques from extreme value statistics have been applied to the task of novelty detection, motivated by the observation that novel events tend to occur in the tails of a probability distribution describing a data class [78].

Alternative approaches to novelty detection include those based on artificial neural networks (ANNs) [79]. ANNs are routinely applied to solving classification problems and novelty detection is a specialised type of classification problem where the purpose is not to recognise the actual class, but simply recognise that the test point originates from some new class. One type of ANN, the novelty filter [80], has been developed for the case of suppressing background noise [81], which is related to the work discussed here, where the novelty filter scheme is similar to the use of sensory signal (only) in our novelty detection scheme. This same novelty detection scheme, that uses the sensory signal, was proposed for the application of noise cancellation in the context of periodic noise many years previously [32] - to our knowledge this link between noise cancellation and novelty filtering/detection has not yet been highlighted.

The statistical and ANN based methods for novelty detection are generally based on offline training using batches of data before implementation online [82]. An offline approach is not suitable for application to autonomous robotics or realistic for biological scenarios, where in both cases the ability to recognise novelty must be constructed online. The inspiration for novelty detection from noise cancellation theory however, provides a sound theoretical framework on which to base online methods for novelty detection, guaranteeing convergence and stability under well-specified conditions [32].

In the context of robotics, an online method for novelty detection inspired by the biological phenomenon of habituation has been developed by Marlsand et al. [83]. That scheme is designed so that often seen stimuli are eventually ignored. The approach contrasts to our scheme, which learns to ignore predictable stimuli and stimuli correlated with motor commands, so that, although contacts are often seen, they are not ignored because they are not predictable. A further development in novelty detection for robotics has been the application of Bayesian decision making, where accumulated evidence leads to a statistical hypothesis test [84]. Our approach is distinct from an evidence accumulation method because it responds rapidly to a single encounter with an object. Hence, our scheme is highly suited to scenarios such as threat and prey detection, where response times must be fast.

### Detection of Novel Vibrissal Contacts in Rat

We have argued (Results) that in a neural counterpart to the signal-processing architecture of Fig 1C the superior colliculus is the most plausible candidate for the comparator (Fig 8). Moreover, the superior colliculus provides climbing fibre input to the cerebellar cortex (via the inferior olive), as required for model-based novelty detection. Anatomical and electrophysiological evidence shows that in rodents the superior colliculus projects via the caudal medial accessory nucleus of the olive to two separate cortical regions [64], the mTOR (medial tecto-olivary recipient) area in vermal zone A1, and the lTOR (lateral tecto-olivo recipient) area in paravermal zone A2 Fig 9).

The mTOR area appears to correspond to the oculomotor vermis as described in cat and primate [64], a region concerned with aspects of eye-movement control such as the calibration of saccadic accuracy [85], [86]. Eye movements are obtained from stimulating this area in rabbits [87], and in rats orienting movements of the head and body may also be involved [63]. Furthermore mossy-inputs connections of the medial TOR area in rat suggest a role in eye- and probably head-movement control (e.g. [64], [88], [89], [90]). It has been argued that orienting in rats is much more influenced by tactile than visual cues (e.g. [91]), and it is of interest that in this context an inaccurate head movement could give rise to an unexpected vibrissal contact. Such a stimulus can in principle be used as an error signal for restoring movement accuracy, as has been proposed for postsaccadic visual signals in primate (e.g. [92], [93]). Current evidence thus supports a role for mTOR in movement control rather than as the location of the hypothesised internal model required for novelty detection.

Much less is known about the lTOR area in zone A2 (e.g. [94]), partly because it has not been described in cat or primate. However, given the suggestion that its climbing fibre input does signal unexpected vibrissal contacts [74], [75], [76], it is a natural candidate for the location of an internal model used for detecting novel vibrissal stimuli. An important question therefore is how far the output and mossy-fibre input connections of this area are consistent with the circuitry required for model-based novelty detection.

#### Connections of Lateral TOR Areas (Zone A2)

*Outputs*. In the robot, the comparator receives an estimate of the noise produced by the robot's own movement from the adaptive filter (Fig 1C). In the neural equivalent of Fig 1C the rat superior colliculus is therefore shown as receiving a projection from the cerebellum (Fig 8). Since almost all the outputs of cerebellar cortex are channelled through the deep cerebellar nuclei, this implies that the superior colliculus should receive inputs from these nuclei. The zonal organisation mentioned above extends to cerebellar output: each cortical zone projects to its own region of the deep cerebellar nuclei, which in turn have distinctive pattern of projections to the rest of the brain. A number of studies have shown that the A2 zone in rat projects to a region of the deep cerebellar nuclei known as the dorsolateral protuberance, part of the fastigial (or medial) nucleus not found in cats or primates [67], [69], [73], [95]. The dorsolateral protuberance sends a projection to the superior colliculus [96]–[97] as required by the model.

**Figure 10:**
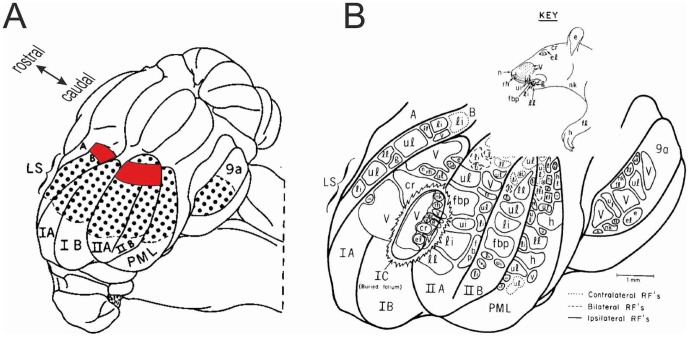
Vibrissal mossy-fibre inputs to lateral TOR areas. A Diagram adapted from Fig 2H of Shambes et al. [99], showing a dorsolateral view of the left posterior cerebellum in rat. The dotted area shows the regions of cerebellar cortex where short latency responses to tactile stimulation can be recorded from the granular layer. Our estimate of the location of lateral TORs 1 and 2 is marked in red. LS lobulus simplex; IA, IB, IIA, IIB crural subdivisions; PML paramedian lobule. B Diagram adapted from Fig 1D of Kassel [88], showing the organisation of the tactile areas illustrated in panel A. Patches of cortex responding to e.g. stimulation of the vibrissae (marked ‘v’, as indicated by the insert titled KEY) are interspersed with patches responding to other regions. This distinctive arrangement is known as 'fractured somatotopy'', and appears to reflect patterns of mossy-fibre collateralisation [108], [131]. The lateral tecto-recipient regions are thought to contain patches responding to vibrissal stimulation, as well as patches responding to stimulation of lips, teeth and perioral regions of skin [63], [100].

*Vibrissal Mossy-Fibre Inputs*. In Fig 1C one of the main inputs to the adaptive filter is a copy of the vibrissal input sent to the comparator. In the adaptive-filter model of the cerebellum, the main filter inputs correspond to mossy fibre signals (Fig 8). Does zone A2 receive vibrissal mossy-fibre inputs? It forms the medial-most part of the extensive tactile area in rat lobules VI and VII first described by Shambes et al. [98], [99], on the basis of mossy-fibre inputs as revealed by granular layer recordings in response to mechanical stimulation of different parts of the body (Fig 10A,B). Difficulties in determining the precise tactile inputs to A2 arise both from uncertainties about the location of its lateral border, and from the way in which inputs from different regions of the body occupy small, intermingled patches (‘fractured somatotopy’). Even so, inspection of the maps obtained by Shambes et al. [98], [99] has suggested that parts of the lateral TOR area do indeed receive vibrissal input [63], [100]. Both response latency data and anatomical findings indicate that some of this vibrissal input to A2 arrives directly from the trigeminal nucleus [98] [90], [101], [102], [103], consistent with the circuit of Fig 8.

*Efference-Copy Mossy-Fibre Inputs*. The second main filter input in Fig 1C is an efference copy of the whisking commands. Whisking in rats is controlled (via the facial nucleus) by a combination of signals from motor cortex, superior colliculus, and sensory cortex area S1 (e.g. [104], [105], [106]). It is known that the medial paravermis of lobules VI and VII (i.e. zone A2) receives vibrissa related mossy-fibre inputs from two of these structures, namely the superior colliculus [88] and area S1 of somatosensory cortex [103], [107], [108]. It may also receive input from vibrissal motor cortex, which projects heavily to the pons [109], and thence probably to lobules VI and VII [110]. However, whether this projection specifically includes the tecto-recipient regions of A2 has yet to be established.

In summary, the connections of cerebellar zone A2 appear to be broadly consistent with those needed for model-based novelty detection of whisker inputs. This consistency provides a basis for further experimental work on the details of the input-output transformations carried out by the TOR areas in this zone.

#### Functions of Lateral TOR Areas

A natural step in investigating these functions would be to record how Purkinje cells in lateral TOR areas respond to vibrissal contact during active whisking. In particular it would be of interest to see whether the response is influenced by whisking and sensory regularity, as predicted by the internal-model architecture as to whisking. However, the question of exactly which cortical areas to record from has yet to be resolved. As indicated in Fig 9, Akaike [62], [63], [72], [100] described two apparently separated TOR regions arranged rostro-caudally in the paravermis from lobule VIa to VII. Brief references are also made by Akaike [63] to a third region in the paramedian lobule (Fig 9). These regions had overlapping but not identical olivary inputs, suggesting functional differences between them [63]. More recent anatomical studies have indicated that the olivary projection to zone A2 has an extremely complex organisation [68], [73], [111], with cortical areas that receive collicular input via the olive intricately interleaved with areas that do not [70].

Recordings from the dorsolateral protuberance (dlp) of the deep cerebellar nuclei (previous section) might help resolve this complexity. The signals sent by this structure to the superior colliculus have not been identified. It has been shown that the circling induced by infusing bicuculline unilaterally into the rat superior colliculus, possibly related to ‘ghost orienting’ (see above), is attenuated by injection of GABA into the deep cerebellar nuclei [112] and that such injections also affect the responses of collicular neurons to stimulation of the vibrissae [113]. However, the cerebello-collicular projection arises from widespread regions of the deep cerebellar nuclei (e.g. [97], [114]), and any contribution of the specific projection from the dlp to these effects has yet to be identified. Moreover, given that this projection is almost certainly excitatory, how it could be subtracted from trigeminal input needs to be investigated. It also needs to be established how far the projections to dlp from zone A2 [67], [95] [69], [73] arise from tecto-olivo-recipient areas in that zone [71], [115].

Finally, the structural complexity of zone A2 might relate to the functional complexity associated with the detection of novel whisker contacts. The architecture of Fig 1C was designed to address the problem of ‘ghost orienting’ in the robot rat. However, improved detection of whisker contacts is also useful for other purposes, such as defence or prey capture. It has been argued previously that the superior colliculus implements a decision-tree about transient stimuli, only the first step of which is whether a stimulus is self-produced [49], [51]. If a transient is judged not to be self-produced, it is then fed into a second stage to determine whether it requires immediate action such as escape or pursuit. Only then does the decision to orient become relevant. Involvement of zone A2 in these multiple stages might account for at least part of its complicated connectivity.

### Implications for Cerebellar Role in Active Sensing

Although there is good evidence that the cerebellum is involved in active tactile sensing [16], [17], [18], [19], [20], the precise nature of its role is not well understood. Detailed circuits indicating how a cerebellar internal-model could be used for noise cancellation have not to our knowledge been proposed In contrast, detailed architectures for noise-cancellation have been described for "cerebellar-like" structures, such as the electrosensory lateral line lobe (ELL) of mormyrid electric fish. We therefore compare the ‘pre-cerebellar’ circuits with the one proposed here for the cerebellum, to indicate the similarities but perhaps more importantly the differences.

#### Noise Cancellation by Cerebellar-Like Structures

**Figure 11:**
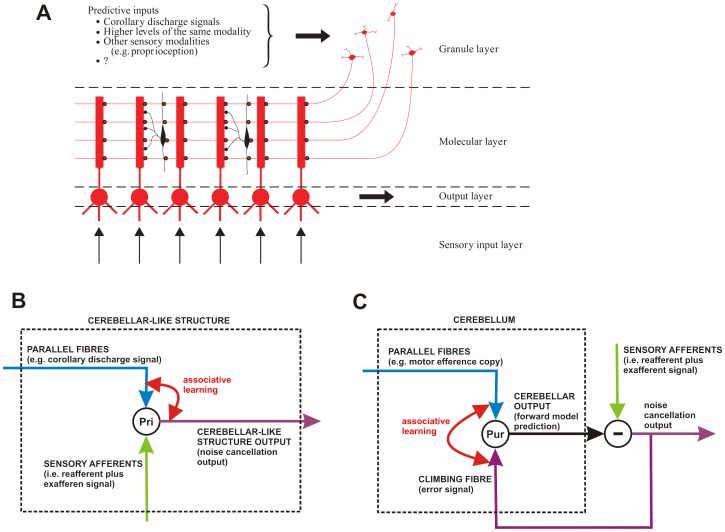
Comparison of cerebellum and cerebellar-like structures. A: Schematic diagram of the cerebellar-like structures in electric fish Fig 2 of [132]. Apical dendrites of principal cells in the output layer receive input from parallel fibres carrying signals such as corollary discharge of the electric organ and proprioceptive signals reporting body movement. Basilar dendrites receive input from the periphery - sensory afferents that carry e.g. electroreceptive information contaminated by reafferent signals. B: Simplified version of panel A to illustrate role of cerebellar-like structures in noise-cancellation. The input pathway via the parallel fibres/apical dendrites is thought to perform the function of a forward model. The forward model prediction is subtracted from the contaminated sensory signals that arrive via the basilar dendrites. The output of the structure is the prediction of the exafferent signal. The principal cell (Pri) thus embodies the complete noise cancellation scheme. Associative learning is driven by correlation between the principal-cell [51] output and the parallel fibre inputs. C: Simplified diagram of the mammalian cerebellum in a hypothesised noise cancellation scheme, drawn to emphasise its relationship with panel B. In contrast to the cerebellar-like structure, the cerebellar output from Purkinje cells (Pur: deep cerebellar nuclei not shown) is the forward model prediction of the reafferent signal. An additional structure is therefore required to act as comparator to predict the exafferent signal, and an additional pathway is required to feed the error signal back to the Purkinje cell to drive associative learning – the climbing fibre.

These cerebellar-like structures adaptively remove self-generated interference from the electroreceptor signal [116], [117], [118]. Principal cells (in the output layer receive (i) corollary-discharge and proprioceptive information from neurons in the granular layer that form synapses with its apical dendrites (Fig 11A), and (ii) direct sensory information from its basal dendrites. The synapses on the apical dendrites are plastic, and their weights are adjusted according to the correlation between the firing of their parent parallel fibre and the firing of the principal cell. The Anti-Hebbian rule for adjusting the weights is similar in form to that used here [119], with the result that the sum of the weighted granule layer inputs comes to form a negative image of the self-generated interference. This is combined with the actual sensory input arriving at the basal dendrites, so that the output of the principal cell forms an estimate of the uncontaminated sensory signal [120], [121]. Overall the operations of cerebellar-like structures bear a striking resemblance to those of adaptive noise-cancelling architectures [122], [123].

However, there is a key difference between the cerebellar (Fig 11C) and cerebellar-like architectures (Fig 11B) in that the output layer cells in cerebellar-like structures embody *both* the adaptive filter *and* the comparator of predicted and observed sensory signal. This arrangement has the advantage that the firing rates of these cells can be used directly as a teaching signal, whereas the more complex arrangement of Fig 8 requires an indirect teaching signal to be conveyed to the cerebellar Purkinje cell by the climbing fibres arising from cells in the inferior olive. "The presence of a climbing fiber is perhaps the critical difference between the cerebellum and cerebellum-like structures" ([117], p.10). But the more complex arrangement has its own advantages. One is that an explicit estimate of sensory interference is available for distribution to appropriate targets in the rest of the brain. A second is that cerebellar output is no longer constrained to act as a teaching signal, so is freed for other purposes such as cancelling interference by moving the sensor in question, as in the vestibulo-ocular reflex [123], [124]. The evolution of new olivary circuitry (Fig 11C) to the basic architecture seen in cerebellar-like structures (Fig 11B) thus enables a great increase in computational flexibility and power.

#### Noise Cancellation by Cerebellum

Previous proposals concerning a possible sensory role for the cerebellum (e.g. [16], [125], [126]) have been made at a more general level than the specific suggestion about model-based novelty detection put forward here. Indeed novelty detection is only one of a number of the sensorimotor competencies required for active sensing (e.g. [8]), and perhaps one of the simpler ones at that. However, such simplicity may prove to be an advantage at this early stage of relating signal-processing theory to the details of cerebellar anatomy and electrophysiology. A plausible assumption is that different sensorimotor competencies are associated with different cerebellar zones, but if so the details of the arrangement are currently very unclear. The specific hypothesis put forward here concerning a role for zone A2 in vibrissal novelty detection is a step towards clarification.

One specific anatomical feature that may be illuminated by the hypothesis concerns overlap between climbing-fibre and granule-cell inputs. As noted earlier, granule-cell input to both paravermis and hemispheres of lobules VI and VII is organised in a distinctive pattern, termed ‘fractured somatotopy’ (Fig 10B). Furthermore, it appears that in some instances individual patches of cerebellar cortex receive climbing-fibre inputs from the same region of the body as their granule-cell fibre inputs [108], [127], [128]. If the internal-model architecture is correct, this would correspond to mossy fibre inputs that convey sensory signals from the whiskers synapsing on granule cells lying within the area of cerebellum that is learning the model. This information would then be conveyed to Purkinje cells and molecular-layer interneurons by the ascending axons of the granule cells. In contrast, by implication, mossy fibres that carry efference-copy signals would synapse with granule cells lying outside this area, and their information would reach the area via parallel fibres. In this arrangement therefore the two mossy-fibre inputs to the cerebellum shown in Fig 8 would use different routes to affect Purkinje cell firing. Whether this corresponds to a special functional significance for past sensory signals in novelty-detection would remain to be investigated.

#### Internal Models and the Cerebellum

It is frequently argued that internal models play a central role in sensory prediction, and that the cerebellum is important for learning such models [2], [5], [6]. However, the detailed mapping of the internal-model architecture onto known biological control circuitry has proved a challenging problem (e.g. [129]). A major stumbling block is that the neural circuitry underpinning many motor-control tasks is extremely complex, especially in the case of limb control where the spinal cord is involved. The detection of novel vibrissal contacts as a preparation for investigating the possible role of the cerebellum in learning internal models has the advantage of not directly involving the spinal cord, being based instead on brainstem and cortical connections about which a great deal is known (e.g. [22]. In addition the novelty detection task is relatively simple, especially when compared with possibilities such as the use of multiple internal models for representing the properties of different tools [6]. Thus, the attempt to understand the detection of novel vibrissal contacts at both computational and implementational levels may significantly illuminate the functions of the cerebellum in forming and accessing internal models.
